# Patient and Carer Understandings and Experiences of Living With Type 2 Diabetes in India

**DOI:** 10.1111/hex.70229

**Published:** 2025-03-15

**Authors:** Rahul Krishna Puvvada, Joanne Marcucci, Clarice Tang, Jency Thomas, Peter Higgs, Ramesh Madhan, Sabrina Gupta

**Affiliations:** ^1^ Department of Microbiology Anatomy Physiology and Pharmacology (MAPP) School of Agriculture Biomedicine and Environment (SABE) La Trobe University Melbourne Victoria Australia; ^2^ Department of Pharmacy Practice JSS College of Pharmacy, JSS Academy of Higher Education and Research Mysuru Karnataka India; ^3^ Department of Public Health School of Psychology and Public Health La Trobe University Melbourne Victoria Australia; ^4^ School of Health Sciences Western Sydney University Campbelltown New South Wales Australia; ^5^ College of Sport, Health and Engineering Victoria University Melbourne Victoria Australia; ^6^ Burnet Institute Melbourne Victoria Australia

**Keywords:** carers, patients, perceptions, qualitative, self‐medication, type 2 diabetes

## Abstract

**Background:**

The prevalence of type 2 diabetes (T2D) is rapidly increasing in India. Evidence suggests that adherence to both pharmacological and nonpharmacological treatment regimens combined with support from carers may help in the optimal management of T2D. However, adherence to treatment and the role of carers is not clearly understood in the management of T2D in India.

**Objective:**

To explore the perceptions of people living with T2D and the role of carers in the management of T2D.

**Methods:**

Semi‐structured face‐to‐face interviews were conducted with people living with T2D in Mysuru, India, and their carers. A total of 22 participants were included, of which 12 were supported by carers. All interviews were conducted in the participant's home, were audio recorded, transcribed verbatim and thematically analyzed.

**Results:**

Two themes were identified (a) the illness journey of people living with T2D; (b) the role of carers in supporting the illness journey of their family members living with T2D. The beliefs and perceptions of people living with T2D impacted their adherence to T2D management. Lack of rapport and open‐ended discussions regarding medication use with doctors are some factors that contributed to self‐medication practices. In addition, there was little trust towards Western medicines, thereby increasing self‐medication of traditional medicines. Carers provided support to their family members in managing T2D, however perceived a sense of powerlessness in their ability to effectively provide support for the management of the disease.

**Conclusion:**

Participants reported non‐adherence to the T2D treatment regimen prescribed by their doctor despite support from carers. There were multiple individual and systemic factors that encouraged self‐medication for people living with T2D. Strategies to garner trust between doctors and patients as well as inclusion of carers during consultations should be considered. This may allow for more open communication and disclosure of self‐medicating and use of traditional forms of medicine.

**Patient Contribution:**

Participants were interviewed by one researcher and contributed to recruitment of additional participants through snowball referral. Participation in the study was voluntary and no financial compensation was provided. Participants also provided feedback to the researcher by reviewing their own interview transcript to ensure clarity.

## 1 Introduction

Type 2 diabetes (T2D) is a chronic health condition that affects the way the body processes blood sugar. Managing T2D often requires pharmacological and nonpharmacological interventions, such as regular exercise, dietary restrictions, and weight reduction [1, 2]. Approximately 90% of individuals with diabetes have T2D, and nearly 80% of these individuals live in low‐ and middle‐income countries, where the prevalence is rising at the fastest rate. Recent estimates show that 212 million people are living with diabetes in India and of this number 85%–95% have T2D [3].

Self‐management is recommended for people living with T2D [4], empowering them to actively participate in their care by adhering to medications, conducting regular blood glucose monitoring, and following exercise and diet recommendations [4, 5]. However, patients may face barriers in adhering to self‐management activities. This impacts care pathways, patient behaviours, and their illness journey due to a disease‐oriented treatment approach [6]. The illness journey involves a series of steps starting from patients' awareness of the disease, management, medications, and their interaction with various stakeholders such as doctors and pharmacists [7]. Understanding the illness journey offers insight into patients' knowledge and experiences of living with T2D [7]. People living in India often have unique cultural and social contexts that influence their T2D illness journey, including traditional beliefs about health, dietary preferences, and social and cultural obligations. Therefore, identifying key aspects of the illness journey that may have the possibility of derailing the patient's disease management will help in prioritizing the treatment options.

Carers play an essential role in the illness journey, particularly in collectivist and interdependent cultures like India. In such cultures, the disease burden may be shared with friends and family members who provide social and emotional support for effective disease management [8]. Studies have shown that providing support through carers encourages people to adopt lifestyle changes and improve their medication adherence and blood glucose levels [9, 10]. However, due to the rapid urbanization and rise of nuclear families in some strata of the Indian society, health outcomes of people living with T2D may be poorer as there can be lack of social, mental, and economic support typically offered by family members in a joint family unit [11]. There is limited knowledge about carers' experiences and perceptions of T2D management and the support they provide. Often the burden on the caregiver and their needs are overlooked by healthcare professionals [12]. Therefore, this study aims to explore the perspectives of people living with T2D in India, their carers and the management of T2D.

## 2 Methods

### 2.1 Study Design

A phenomenological qualitative research approach was used to explore the experiences and perceptions of both people living with T2D and their carers in the management of T2D. The methodology and findings are presented according to the consolidated criteria for reporting qualitative research (COREQ) guidelines [13]. The trustworthiness of the study was enhanced using Lincoln and Guba's evaluation criteria [14].

### 2.2 Study Participants and Recruitment

This study was conducted between January 2021 and February 2022 in Mysuru, a city in the south of India, with a population of approximately three million people [15]. The sample included any adult living with T2D. Carers were defined as individuals who assist those with chronic illness by managing medications, supporting daily activities, and attending doctor appointments [16]. The participants were recruited via research information flyers placed at diabetes outpatient clinics at Jagadguru Sri Shivarathreeshwara Hospital, Mysuru, and through a snowball sampling method. Potential participants expressed interest by contacting the investigator (Rahul Krishna Puvvada) who screened the participants for inclusion in the study and explained the participant information and consent process.

### 2.3 Data Collection

A mutually convenient date, time, and place to conduct the interview was discussed, and participants all chose their own homes. A signed informed consent form and participant demographics form was obtained from all participants before initiating the interview. During the interview, the participants were asked if they have any carers involved in the management of T2D. For participants with carers, they were invited to participate in an interview after the interview with the person living with T2D. Each participant was assigned a unique code (‘Pt‐01’ for people with T2D and ‘Ca‐01’ for carers) to maintain confidentiality. Codes reflect the order of interviews.

The interview guide was developed drawing on these studies [10, 17, 18, 19, 20] and through contributions of the research team who all have experience in conducting qualitative and diabetes related studies in India. The interview guide consisted of two sets of semi‐structured questions and prompts. The first focused on perceptions of T2D management strategies and interactions with healthcare professionals from the perspective of individuals living with T2D. The second was carers' understandings of T2D, their perceptions, and experiences in managing their family member's T2D. Rahul Krishna Puvvada, an experienced qualitative researcher, conducted the interviews. Being part of the local community may have enhanced the trust and rapport with the participants. The interviews were conducted until data saturation was obtained, defined as the point when no additional information or themes emerged after two consecutive interviews [21]. This ensured thorough thematic exploration, confirming that further data collection would not yield additional relevant information. All the interviews were transcribed verbatim by Rahul Krishna Puvvada and the transcripts were sent to participants for member checking which enhanced credibility and confirmability [22].

### 2.4 Data Analysis

All the transcripts were uploaded to NVIVO‐12 (QSR international) for assistance with coding the interviews [23]. This study used Braun and Clarke's step‐by‐step guide for performing reflexive thematic analysis [24]. All transcripts were coded independently by Rahul Krishna Puvvada and Joanne Marcucci. After completion of the initial coding, the two researchers met to analyze the coded data and identify the data points that have the potential to form themes and sub‐themes. Later, the themes, sub‐themes, and respective codes were refined to achieve coherence. The final consensus on themes and sub‐themes was obtained after meeting with the other research members who provided perspectives informed by their prior research experience.

## 3 Results

A total of 30 people living with T2D expressed interest in the study, but only 22 people were successfully recruited. Of these, 12 had a carer who also completed the interviews. The findings are presented in two sections: *People living with T2D* and *Carers*.

### 3.1 People Living With T2D

Most participants were male [14 (64%)], and the overall mean age of participants was 64.7 ± 8.9 years. They primarily used oral antidiabetic medications [17 (77%)] with others using a combination of oral medications and insulin [5 (23%)]. Most lived in urban areas [18 (82%)] compared with rural areas [4 (18%)]. See Table 1 for demographic details.

**Table 1:** Demographic details of people living with T2D.

Participant characteristics	People living with T2D (*N* = 22) n%
Sex	
Male	14 (64)
Female	8 (36)
Mean age ± SD (years)	64.7 ± 8.9
Area of residence	
Urban	18 (82)
Rural	4 (18)
Years living with T2D (range 1.5–30 years)	
≤ 10 years	12 (54)
11–20	7 (32)
21–30	3 (14)
Age of onset of T2D	
20–30	1 (4)
31–40	3 (14)
41–50	6 (27)
51–60	9 (41)
61–70	3 (14)
Type of antidiabetic medications	
Only oral medications	17 (77)
Only insulin	0
Combination of both oral medications and insulin	5 (23)

The overarching theme was *the illness journey of people living with T2D*. The subthemes are presented below in Table 2. Interviews lasted an average of 40 min.

**Table 2:** Themes and sub‐themes from the people living with T2D interviews.

Sub‐themes	Theme
T2D is perceived as common but not serious	The illness journey of people living with T2D
Difficulty in integrating lifestyle modifications into daily life	
Participants believed T2D can be managed without Western medications	
Perceptions regarding engagement with doctors in the management of T2D	
Transactional relationship with pharmacists	
Carers' support in T2D management	

### 3.2 The Illness Journey of People Living With T2D

Participants described their illness journey, including symptoms, diagnosis, and management. They discussed their beliefs about both Western and traditional medications and their impact on T2D self‐management.

#### 3.2.1 T2D Is Perceived as Common but Not Serious

Participants often dismissed T2D as insignificant until diagnosis. Symptoms like dizziness and tiredness prompted 12 participants to seek medical advice, while 10 were diagnosed incidentally.

‘I used to feel tiredness, frequently hunger, and dizziness when I am doing my work. Never I experienced like that, and I don't have any idea why it was happening to me like that. After doing tests, doctor said that I have diabetes’.

(Pt‐15, Male)

‘When I had plan to go to US, that time they need full body check‐up that time I came to know that sugar was above 400 and doctor said I have diabetes’.

(Pt‐16, Female)

Upon T2D diagnosis, many participants (n = 13) were shocked. However, some participants (n = 9) were more accepting, viewing T2D as a common, lifelong condition requiring lifestyle modifications.

‘I was stunned, initially I was stunned when the doctor said I have diabetes. No one in my family had diabetes. We were healthy, but it will be there with me for lifelong. So, I thought to accept it and got used to it’.

(Pt‐12, Male)

‘When doctor said I have diabetes, I thought it is common. All over the world, it is common. One day BP [blood pressure] will come, diabetes will come. It is also a part of life as age increases. I did not panic’.

(Pt‐05, Male)

#### 3.2.2 Difficulty in Integrating Lifestyle Modifications Into Daily Life

All participants (*n* = 22) were aware of the diet requirements but found them challenging to implement, particularly as food was prepared for the entire family. Rice was the most preferred food, making it difficult to exclude. However, participants believed reducing rice portions and replacing it with chapatis (Indian flatbread) helped control blood glucose levels.

‘Food is prepared commonly for all. So, I eat everything. But without rice I cannot eat. It is common in our food. So, I reduce rice and [have] two chapati. My sugars are controlled like this’.

(Pt‐15, Male)

Most participants (*n* = 20) reported regular physical activity, such as walking for 30–40 min but some had their exercise routines disrupted during the COVID‐19 lockdown.

‘I go for walking regularly. But due to COVID‐19 lockdown, I avoided to go out. So, I walked in my home but for less time. Now everything is back to normal’.

(Pt‐10, Female)

Additionally, an issue pertaining to insulin storage was raised by one participant who highlighted the difficulty in maintaining the required temperature without access to a fridge.

‘We will keep it in fridge at neighbour's house. But I have to take twice in a day. So, I will keep it in cold water. I will use it and by night I will keep it in fridge. Doctor told us to keep in fridge only’.

(Pt‐06, Female).

#### 3.2.3 Participants Believed T2D Can Be Managed Without Western Medications

Fifteen participants preferred traditional medications over Western medications, perceiving them as free from adverse effects, and adjusted their prescribed medication accordingly. They also mentioned that they self‐medicated with traditional medicine at least once during their illness journey.

‘There are no side effects in traditional medications. So, I believe in traditional medications. I haven't experienced any side effects. But Western medications have side effects. I have experienced it [itchy legs] personally. So, I will take Western medications at low doses and take more doses of traditional medications’.

(Pt‐01, Male)

Some participants (*n* = 7) mentioned that information from friends and family members and/or reading newspapers led them to perceive that T2D could solely be managed by regular physical activity if their blood glucose levels were within normal limits. Participants engaged in these activities to avoid taking Western medications. In some cases, the participants (*n* = 2) were able to control their blood glucose levels, while in others there was less control (*n* = 5).

‘I read in newspaper that exercise is important in diabetes. So, I thought if my sugars are within normal then why take medications? So, for 1 year I did not take the medicines. I am doing walking, some household work daily. I am controlling like this only. I did not show to doctor again’.

(Pt‐18, Female)

‘For 1.5 years my blood sugars were in control. With just that two tablets. My friends told me exercise and walking are more important than medications. I was pretty much fine, with my exercise and walking. So, I just gave a try of not taking the medication but trying to control it [blood glucose] with my walking and exercises. But it did not work. My sugars increased’.

(Pt‐04, Male)

#### 3.2.4 Perceptions Regarding Engagement With Doctors in the Management of T2D

All participants desired to maintain positive relationships with doctors. More than half of the participants (*n* = 12) reported informing their doctors about their self‐medication practices or discontinuing Western medications. They mentioned that their doctors insisted they stop taking traditional medications and were re‐prescribed Western medications.

‘Once I stopped [Western] medications and tried ayurvedic [traditional] medications. I wanted to see how it [traditional medications] works. When I went to meet doctor, I informed him that I am taking ayurvedic medications. He [doctor] said sugars are not in control and not to stop [Western] medications or else my sugars may increase more. He told me to stop ayurvedic medications and prescribed new [Western] medications and insulin’.

(Pt‐06, Female)

Due to the fear of negative responses toward the use of traditional medications from doctors, some participants (*n* = 10) were not willing to disclose their self‐medication practices during appointments.

‘Sometimes after taking [Western] medications, I start sweating. So, I stopped taking it and took some ayurvedic [traditional] tablets. Recently I visited him [doctor]. He said my sugars are high and increased the dose. But I did not inform him that I did not take medications. I thought he will scold me’.

(Pt‐15, Male)

Another aspect most participants (*n* = 20) expressed concern over was doctors' perceived prescription practices. They mentioned that doctors prescribed branded medications, stating they were more effective than lower‐cost generic medications.

‘I asked my doctor it's been many days I am using these [branded] medications. It is a bit costly. I asked him to give less costly medications. But he said, your health is good now and blood sugars are controlling well with these [branded] medications so he asked me to continue the same’.

(Pt‐11, Male)

Some participants (*n* = 10) said they used generic medications, which were available through the government‐funded Jan Aushadi pharmacies that provide medications at subsidized costs. These participants expressed concerns about disclosing their use of generic medications as they believed doctors would switch them to branded and more expensive medications.

‘He did not suggest me to take Jan Aushadhi [generic] medications. I took it because of less cost. I won't tell him also. He may ask me to change to branded medications if I tell him. Anyways my sugars are good now’.

(Pt‐15, Male)

Most participants (*n* = 14) said their doctors regularly checked their blood glucose levels and suggested medication, or dose alterations based on the blood glucose readings. They also mentioned that doctors educated them about the disease and management strategies.

‘Whenever my sugar increases, she [doctor] says not to worry and because of eating too many sweets, it increased. Then she will increase my tablet dose. Again, when I go to her…she will do the blood test again and reduce the dose if my sugars are normal’.

(Pt‐21, Female)

‘My doctor told me if I miss taking medications, then sugars will increase. Also, he said that insulin is better than tablets, because tablets may have some side effects like kidney failure but insulin if taken in right way and dose it won't cause any side effects’.

(Pt‐04, Male)

One participant said her doctor applauded her for maintaining her blood glucose levels within the normal limits.

‘Whenever sugar levels are in control, she used to praise me’.

(Pt‐21, Female)

#### 3.2.5 Transactional Relationship With Pharmacists

In contrast to the relationship with doctors, all participants (*n* = 22) mentioned that they did not have a long‐standing professional relationship with pharmacists. Their concerns regarding medications were answered by their doctor so they felt no need to raise any concerns with pharmacists. They perceived pharmacists' roles as limited to dispensing medications. Most participants (*n* = 15) shared the name of their medications with the pharmacist to obtain their medications without a prescription. The remaining participants (*n* = 7) said they either showed their used medication strip, an expired prescription, or relied on being recognized as regular customers to purchase their medications.

‘Every pharmacist will say the same thing. Take as advised by the doctor. He also sees the prescription and tell. See, in the prescription itself doctor will write 1‐0‐1, 0‐0‐1, 1‐1‐0 [reflecting whether to take medications during breakfast, lunch and/or dinner] that clearly indicates when to take, how to take as per the prescription. There is no question of asking the pharmacist also’.

(Pt‐05, Male)

‘Prescription is not needed. I am using daily, so I know the name of the medications. Whichever pharmacy I go, I will ask…medications. They [pharmacists] just have to give medications’.

(Pt‐11, Male)

‘I just simply show my medication strip or doctor's prescription, and he [pharmacist] will give. There is no discussion at that place [pharmacy]. I am using these regularly. So, he knows me. He will understand by seeing my face’.

(Pt‐02, Male)

#### 3.2.6 Carers' Support in T2D Management

Many participants (*n* = 12) mentioned receiving active support from their family members (carers) for visiting the doctor, purchasing medications, and managing T2D at home.

‘Sometimes I may forget to take medications or lazy to go for walking or feel like to eat more rice or other junk food. But my wife will see everything I am doing and controls me. I think because of her my sugar levels are managing very well’.

(Pt‐09, Male)

Other participants (*n* = 10) said they self‐managed T2D without carers.

‘I take medicine regularly. I am independent. I don't want to be dependent on anyone. Because I can do it on my own’.

(Pt‐03, Male)

### 3.3 Perceptions and Experiences of Carers in Managing Their Family Members' T2D

The results from the carers (*n* = 12) are presented in this section. Most of the carers were male, and the overall mean age of participants was 61.8 ± 8.8 years. Most carers were partners (husband, *n* = 6; wife, *n* = 5) with one being a son (Table 3). The themes and sub‐themes emerging from the interviews are presented in Table 4.

**Table 3:** Demographic details of carers.

Characteristics	Carers (*N* = 12) n%
Sex	
Male	7 (58)
Female	5 (42)
Mean age ± SD (years)	61.8 ± 8.8
Area of residence	
Urban	18 (82)
Rural	4 (18)
Relationship to the person living with T2D	
Husband	6 (50)
Wife	5 (42)
Son	1 (8)

**Table 4:** Themes and sub‐themes from the carer's interviews.

Sub‐themes	Theme
Carers acquired knowledge and understanding of T2D management	The role of carers in the illness journey of people living with T2D
Carers felt despondent and powerless in influencing their family member's T2D management	
Carers' perceptions regarding services provided by doctors	

### 3.4 The Role of Carers in the Illness Journey of People Living With T2D

The overarching theme was *the role of carers in the illness journey of people living with T2D*. Carers supported the management of their family members' T2D by reinforcing medication adherence, restricting food intake, and encouraging physical activity. Despite these efforts, carers expressed concerns about their family members' behaviour and their unwillingness to adhere to the diabetes management plan prescribed by the doctor.

#### 3.4.1 Carers Acquired Knowledge and Understanding of T2D Management

When asked about their understanding of T2D, all carers (*n* = 12) said they were unaware of the condition until their family members were diagnosed. After the diagnosis, they made efforts to learn about the disease either from their doctor or by reading newspapers. They understood that managing T2D involves both pharmacological and nonpharmacological interventions.

‘Before I was not knowing about diabetes. After my husband got it, I got involved. I asked my doctor and even I read some articles and newspapers where they say about diabetes. I understood how to manage it. What will happen if we neglect it. It causes weakness, weight loss and sweating. That is how I understood it is painful. It requires major changes in the lifestyle of an individual. We should reduce weight, do exercise, restrict the food intake and take medications regularly’.

(Ca‐09, Female)

#### 3.4.2 Carers Felt Despondent and Powerless in Influencing Their Family Member's T2D Management

All carers (*n* = 12) reported actively managing their family member's T2D by reminding them to take medications, exercise, and maintain a proper diet.

‘I advise her not to eat too much of rice or sweets, go for walking, eat food at proper time and take medications… In my home, we will ask each other about their medications or food. Sometimes she will delay taking tablets. At that time, I will tell her to take medications as soon as possible’.

(Ca‐21, Male)

While a few carers (*n* = 7) initially prepared food according to the doctor's dietary advice, the food preferences of other family members created the challenge of cooking separate meals for the patient and the rest of the family. As a result, they encouraged their family members with T2D to reduce their intake of carbohydrate‐rich foods like rice and sweets.

‘I used to prepare food that is good for diabetes people. But if person in house likes it, others don't like it. I cannot prepare separate food for all of them. If I prepare anything new, if they don't like it then I only should eat it. I cannot eat same thing for more days. So, I stopped preparing it. But I will tell to reduce the rice intake and have sugarless tea or coffee. So, like that I will restrict [food]’.

(Ca‐22 Female)

Some carers (*n* = 5) expressed disappointment at the lack of adherence to diabetes management advice from doctors, despite continuous reminders.

‘He will decide on his own. He will not listen to me. He won't restrict his diet. He will only decide which medications are best for him. I got tired telling him. He says I don't know about diabetes. So, I stopped telling him’.

(Ca‐14, Female)

#### 3.4.3 Carers' Perceptions Regarding Services Provided by Doctors

All carers (*n* = 12) accompanied their family members to doctor visits and had mixed responses about these consultations. Some carers (*n* = 5) remarked that they were satisfied with their doctor.

‘In phone call also, our doctor will respond very well. She will tell what all to be taken, how many days and all. She is very good and close to our family’.

(Ca‐21, Male)

Many carers (*n* = 7) were critical of the services provided by doctors. These carers expected more education about the disease and necessary precautions.

‘When we go to them [doctor] for a problem, they should have the patience to explain about the problem in detail. They should explain why and what are the reasons for this problem and what precautions we should take.

(Ca‐01, Female)’.

They mentioned that doctors changed medications based on changes in blood glucose levels without educating them about lifestyle changes.

‘They will ask for symptoms and blood glucose levels and they will write a tablet. That's all. What are the reasons for getting that problem, what we should do, what precautions we should take, all those things they don't say’.

(Ca‐01, Female)

Only two participants dissatisfied with their doctor's advice mentioned that they would change doctors.

‘Our previous doctor did not explain much about diabetes… So, I and my husband decided to change doctor. Current doctor is good to us. He will explain clearly what to do, type of food take and exercise’.

(Ca‐16, Female)

One participant continued visiting the same doctor despite dissatisfaction with the services.

We don't want to search for a new doctor now. Our doctor is very close to my house. So, we are continuing with him only.

(Ca‐07, Male)

## 4 Discussion

This qualitative study explored how people living with T2D and their carers experienced and perceived the T2D illness journey, from diagnosis (shock vs. acceptance) to management, and how they understood their new identity as patients through relationships with doctors and pharmacists. Participants living with T2D in this study had greater trust in traditional remedies than in doctors trained in Western models of healthcare. Despite mixed evidence on the effectiveness of traditional medicine for diabetes management, concerns about efficacy, side effects, and interactions arise [25]. Nevertheless, the cultural familiarity and accessibility of these therapies often make them the preferred option. Many participants disclosed that they would not share their self‐medication choices and actions, fearing a patronizing response from their doctor. The results suggest that the illness experience of patients is culturally constructed and discordant with the beliefs held by doctors. Further, a doctor‐centric approach primarily focusing on disease treatment, with little regard for the illness experience, may affect patient adherence and satisfaction with care. In a populous country like India, low doctor‐to‐patient ratio and frequent appointment intervals could also contribute to limiting duration of consultation times [6]. These factors would hinder rapport restricting doctors' understandings of patients' perspectives which affects establishment of trust.

The findings suggest that trust between patients and healthcare professionals, particularly doctors, is critical in managing T2D. Trust between patients and doctors in India has eroded significantly in recent years due to systemic failures and regulatory issues [26]. Factors influencing trust include doctor behaviour, empathy, personal involvement, and cultural competence [27]. To rebuild trust, a balanced approach is needed, focusing on strong regulation of healthcare institutions and nurturing interpersonal relationships between doctors and patients [26]. Strategies should consider addressing the doctor‐to‐patient ratio through expanding the workforce and addressing communication challenges to improve the perceived quality of care.

The lack of exchange of health information between doctor and patient may affect care provision due to a mismatch in doctors' and patients' expectations [28]. An example of this was demonstrated through advice regarding storage and temperature control of insulin. According to the International Diabetes Federation guidelines, insulin must be stored between 2°C and 8°C [29]. Doctors' advice must consider patients' access to resources, such as refrigerators, and options for maintaining insulin temperature, such as using earthen pots filled with water [30]. Efforts to build trust and increase rapport during clinical consultations are essential for gaining a comprehensive picture of patients' illness experiences.

Similarly, the results suggest that the relationship between pharmacists and participants was less than ideal, with participants describing it as superficial and transactional. This may be due to a widespread culture of pharmacies being managed by non‐qualified drug retailers lacking pharmaceutical care skills, as well as the lack of enforcement of pharmaceutical regulations in India [31, 32]. Pharmacists are the third‐largest healthcare providers after doctors and nurses in India and are readily available to patients without any prior appointments [33]. In a country facing an epidemic of T2D, all available resources must be optimized, and pharmacists could play an essential role in addressing this growing health issue. This readily accessible and informed workforce, alongside stringent implementation of pharmaceutical policies, must be considered in the fight against T2D to help patients have a better illness experience.

Health professionals can adopt a behavioural change approach to facilitate improved self‐management among people with T2D in India. Addressing behavioural changes can help identify individual facilitators and barriers to adapting lifestyle changes, including adherence to medications, diet, and physical activity [32]. The COM‐B framework for behaviour change helps recognize social and psychological factors influencing patients' adherence to disease management [34, 35]. The COM‐B model comprises three key elements: C for Capability, O for Opportunity, and M for Motivation, which can change B for Behaviour [34, 35]. It suggests that understanding a patient's capability to manage disease involves assessing their knowledge of the disease, skills for managing it, and providing opportunities by creating a favourable environment for exchange of disease management information between doctors and patients. Furthermore, considerations of collectivist culture and recognizing the key role carers play in disease management can motivate behavior change and improve disease management [34, 35].

Building on insights from the COM‐B model, carers played a significant role in the illness experience of T2D for more than half of the participants. They enhanced patients' capabilities by helping them understand and manage their condition post‐diagnosis. This enabled them to create opportunities for better disease management by accompanying family members during follow‐up visits to discuss disease progression. Additionally, carers motivated patients by providing reminders for medications, diet, and physical exercise, and encouraging self‐care practices. This was supported by previous studies conducted both internationally and in India, which found that carers' long‐term perspective on the illness experience enabled them to provide social and emotional support, contributing to better disease management [11, 36, 37].

However, some carers felt despondent about their family members not adhering to the medical advice from health professionals. Differing perceptions about diabetes management complicate caregiving. Lack of acknowledgement and differing perceptions of disease management, as well as their position within the family structure may affect the disease management support carers can offer [38, 39]. To better support carers, healthcare professionals should actively identify and assess their needs, considering both generic, disease specific as well as societal and gender norms and expectations that may influence caregiving [40]. Future research should explore the complexity of collectivist cultures and the dominant social and cultural norms. For example, developing strategies to support carers' needs in these contexts.

## 5 Strengths and Limitations

This is the first study conducted in India including both people living with T2D and their carers to understand their perceptions of T2D management. Given India's diversity, focusing on a sample from a small city makes these results particularly relevant to that context, offering a rich, nuanced understanding. This localized approach, combined with the qualitative nature of the study, offers unique insights into the importance of context‐specific research. However, given the cultural and societal variations across different regions of India, these findings may not be generalizable to other regions. Snowball sampling was effective for reaching interconnected participants within the community, but it may over‐represent certain types of participants, introducing bias that could affect the generalizability of the findings. Being interviewed by a local may have enhanced rapport and trustworthiness; however, the subjective nature of qualitative research means that the researcher's perspectives, interpretations, and interactions could influence data collection and analysis.

## 6 Conclusion

This study provides insights into the perceptions of people living with T2D and the barriers involved in managing the condition. It found a discordance between how individuals with T2D experience their illness and their perceptions of their doctors' views on its management. Perceived side effects of Western medications, greater trust in traditional remedies, doctors' lack of understanding of individual patient circumstances, and insufficient pharmaceutical regulations often lead to self‐medication practices for T2D management. Clinical care will be more effective when both disease and illness are treated together. Explanatory models should focus on key points requiring patient education, clear clinical explanations, and improved communication between doctors and patients. Future research should explore the role and effectiveness of traditional medicine in T2D management, particularly given patients' strong trust in these remedies. However, integration into patient‐centred care models should be guided by scientific evidence to ensure safety and efficacy. Investigating evidence‐based approaches to incorporating traditional practices may enhance health outcomes through culturally sensitive care. Given the critical role of carers in supporting patients, their inclusion in consultations is essential. Healthcare professionals should also assess carers' needs, acknowledge their contributions, and raise awareness of available support services. These steps can help address behaviours such as self‐medication by building trust and rapport with both patients and carers, leading to improved management and better health outcomes.

## Author Contributions

**Rahul Krishna Puvvada:** conceptualization, methodology, investigation, formal analysis, visualization, writing – original draft, project administration. **Joanne Marcucci:** formal analysis, writing – review and editing, visualization. **Clarice Tang:** conceptualization; methodology; formal analysis; writing – review and editing, supervision. **Jency Thomas:** conceptualization, methodology, formal analysis, writing – review and editing, supervision. **Peter Higgs:** conceptualization, writing – review and editing. **Ramesh Madhan:** resources. **Sabrina Gupta:** conceptualization, methodology, formal analysis, writing – review and editing, project administration,supervision.

## Ethics Statement

Ethics approval was received from the Human Ethics Committee of La Trobe University, Australia (HEC19227) and Jagadguru Sri Shivarathreeshwara Academy of Higher Education & Research (JSS AHER), Mysuru, India (JSSMC/IEC/1107/10NCT/2019–20).

## Conflicts of Interest

The authors declare no conflicts of interest.

## Data Availability

The authors have nothing to report.

## References

[1] N. G. Forouhi , A. Misra , V. Mohan , R. Taylor , and W. Yancy , “Dietary and Nutritional Approaches for Prevention and Management of Type 2 Diabetes,” BMJ (London) 361 (2018): k2234, 10.1136/bmj.k2234. PMC5998736 29898883

[2] J. J. Marín‐Peñalver , I. Martín‐Timón , C. Sevillano‐Collantes , and F. J. Cañizo‐Gómez , “Update on the Treatment of Type 2 Diabetes Mellitus,” World Journal of Diabetes 7, no. 17 (2016): 354–395, 10.4239/wjd.v7.i17.354. 27660695 PMC5027002

[3] B. Zhou , A. W. Rayner , E. W. Gregg , et al, “Worldwide Trends in Diabetes Prevalence and Treatment From 1990 to 2022: A Pooled Analysis of 1108 Population‐Representative Studies With 141 Million Participants,” Lancet 404 (2024): 2077–2093. 39549716 10.1016/S0140-6736(24)02317-1 PMC7616842

[4] ADA ., “Standards of Medical Care in Diabetes—2014,” Diabetes Care 37, no. Suppl_1 (2013): S14–S80, 10.2337/dc14-S014. 24357209

[5] A. N. van Smoorenburg , D. F. L. Hertroijs , T. Dekkers , A. M. J. Elissen , and M. Melles , “Patients' Perspective on Self‐Management: Type 2 Diabetes in Daily Life,” BMC Health Services Research 19, no. 1 (2019): 605, 10.1186/s12913-019-4384-7. 31462220 PMC6714441

[6] A. Kleinman , L. Eisenberg , and B. Good , “Culture, Illness, and Care,” Annals of Internal Medicine 88, no. 2 (1978): 251–258, 10.7326/0003-4819-88-2-251. 626456

[7] R. Devi , K. Kanitkar , R. Narendhar , K. Sehmi , and K. Subramaniam , “A Narrative Review of the Patient Journey Through the Lens of Non‐Communicable Diseases in Low‐ and Middle‐Income Countries,” Advances in Therapy 37, no. 12 (2020): 4808–4830, 10.1007/s12325-020-01519-3. 33052560 PMC7553852

[8] R. Chadda and K. Deb , “Indian Family Systems, Collectivistic Society and Psychotherapy,” Indian Journal of Psychiatry 55, no. Suppl 2 (2013): 299, 10.4103/0019-5545.105555. PMC3705700 23858272

[9] R. A. Pamungkas , K. Chamroonsawasdi , and P. Vatanasomboon , “A Systematic Review: Family Support Integrated With Diabetes Self‐Management Among Uncontrolled Type II Diabetes Mellitus Patients,” Behavioral Sciences (Basel, Switzerland) 7, no. 3 (2017): 62, 10.3390/bs7030062. 28914815 PMC5618070

[10] A. A. Baig , A. Benitez , M. T. Quinn , and D. L. Burnet , “Family Interventions to Improve Diabetes Outcomes for Adults,” Annals of the New York Academy of Sciences 1353, no. 1 (2015): 89–112, 10.1111/nyas.12844. 26250784 PMC4624026

[11] S. Ravi , S. Kumar , and V. Gopichandran , “Do Supportive Family Behaviors Promote Diabetes Self‐Management in Resource Limited Urban Settings? A Cross Sectional Study,” BMC Public Health 18, no. 1 (2018): 826, 10.1186/s12889-018-5766-1. 29973181 PMC6031108

[12] L. Newson , J. E. Brown , and S. Dugdale , “Being the Supporter: An Interpretative Phenomenological Analysis of the Role of Caregivers in the Self‐Management of Type 2 Diabetes,” Psychology & Health 40, no. 3 (2023): 377, 10.1080/08870446.2023.2231004. 37394809

[13] A. Tong , P. Sainsbury , and J. Craig , “Consolidated Criteria for Reporting Qualitative Research (Coreq): A 32‐item Checklist for Interviews and Focus Groups,” International Journal for Quality in Health Care 19, no. 6 (2007): 349–357, 10.1093/intqhc/mzm042. 17872937

[14] Y. S. Lincoln and E. G. Guba , Naturalistic Inquiryeds (Sage Publications, 1985).

[15] District Administration Mysuru . Mysuru District Census. [cited January 04, 2022], https://mysore.nic.in/en/?csrf_nonce=7a5294e847&_wp_http_referer=%2Fen%2F&s=census.

[16] National Health Service (NHS) . Who is Considered a Carer? [cited June 02, 2022], https://www.england.nhs.uk/commissioning/comm-carers/carers/.

[17] R. K. Puvvada , S. Gupta , C. Y. Tang , et al., “Factors Affecting Self‐Medication Practices Among People Living With Type 2 Diabetes in India‐ A Systematic Review,” Metabolism Open 9 (2021): 100073, 10.1016/j.metop.2020.100073. 33364596 PMC7753190

[18] A. Ahmad , M. U. Khan , and P. Aslani , “A Qualitative Study on Medication Taking Behaviour Among People With Diabetes in Australia,” Frontiers in Pharmacology 12 (2021): e693748, 10.3389/fphar.2021.693748. PMC8488297 34616293

[19] M. Deepa , A. Bhansali , R. Anjana , et al., “Knowledge and Awareness of Diabetes in Urban and Rural India: The Indian Council of Medical Research India Diabetes Study (Phase I): Indian Council of Medical Research India Diabetes 4,” Indian Journal of Endocrinology and Metabolism 18, no. 3 (2014): 379–385, 10.4103/2230-8210.131191. 24944935 PMC4056139

[20] A. Koipuram , S. Carroll , Z. Punthakee , and D. Sherifali , “Diabetes Knowledge, Risk Perception, and Quality of Life Among South Asian Caregivers in Young Adulthood,” BMJ Open Diabetes Research & Care 8, no. 2 (2020): e001268, 10.1136/bmjdrc-2020-001268. PMC7654120 33168524

[21] P. Fusch and L. Ness , “Are We There Yet? Data Saturation in Qualitative Research,” Qualitative report 20, no. 9 (2015): 1408, 10.46743/2160-3715/2015.2281.

[22] L. Birt , S. Scott , D. Cavers , C. Campbell , and F. Walter , “Member Checking: A Tool to Enhance Trustworthiness or Merely a Nod to Validation?,” Qualitative Health Research 26, no. 13 (2016): 1802–1811, 10.1177/1049732316654870. 27340178

[23] QSR International . NVIVO Qualitative Data Analysis Software. 2021.

[24] V. Braun and V. Clarke , “Using Thematic Analysis in Psychology,” Qualitative Research in Psychology 3, no. 2 (2006): 77–101, 10.1191/1478088706qp063oa.

[25] D. V. Chandra , P. Yadav , and V. Raghuvanshi Diabetes and Ethnomedicine: A Comprehensive Review of Scientific Literature on Traditional Medical Practices. 8, 2 (2023):01–17.

[26] S. Kane and M. Calnan , “Erosion of Trust in the Medical Profession in India: Time for Doctors to Act,” International Journal of Health Policy and Management 6, no. 1 (2017): 5–8. 28005537 10.15171/ijhpm.2016.143 PMC5193507

[27] V. Gopichandran and S. K. Chetlapalli , “Factors Influencing Trust in Doctors: A Community Segmentation Strategy for Quality Improvement in Healthcare,” BMJ Open 3, no. 12 (2013): e004115. 10.1136/bmjopen-2013-004115 PMC3855707 24302512

[28] J. J. Palmieri and T. A. Stern , “Lies in the Doctor‐Patient Relationship,” Primary Care Companion to The Journal of Clinical Psychiatry 11, no. 4 (2009): 163–168, 10.4088/PCC.09r00780. PMC2736034 19750068

[29] International Diabetes Federation . Insulin Storage and Management Guidelines. 2023 [cited 2024 Sep 10], https://idf.org/europe/media/uploads/sites/2/2023/06/IDF-Insulin-Storage-Doc-PRINT-VERSION-3mm-bleed-201119-FINAL.pdf.

[30] S. Kalra and B. Kalra , “Storage of Insulin in Rural Areas,” Journal of Academy of Medical Sciences 2, no. 2 (2012): 88–89, 10.4103/2249-4855.118669.

[31] R. Soumya , V. Devarashetty , C. Jayanthi , and M. Sushma , “Drug Dispensing Practices at Pharmacies in Bengaluru: A Cross‐Sectional Study,” Indian Journal of Pharmacology 48, no. 4 (2016): 360–364, 10.4103/0253-7613.186204. 27756944 PMC4980921

[32] S. C. Basak and D. Sathyanarayana , “Community Pharmacy Practice in India: Past, Present and Future,” Southern Med Review 2, no. 1 (2009): 11–14. 23093872 PMC3471162

[33] T. W. Xuan Hao Chan Global Pharmacy Workforce and Migration Report: A Call for Action,. 2006 [cited 2022 26 May], https://www.fip.org/files/fip/publications/PharmacyWorkforceMigration.pdf.

[34] P. Mishra , A. S. Vamadevan , A. Roy , et al., “Exploring Barriers to Medication Adherence Using Com‐B Model of Behaviour Among Patients With Cardiovascular Diseases in Low‐And Middle‐Income Countries: A Qualitative Study,” Patient Preference and Adherence 15 (2021): 1359–1371, 10.2147/PPA.S285442. 34188453 PMC8236251

[35] R. West and S. Michie , “A Brief Introduction to the COM‐B Model of Behaviour and the Prime Theory of Motivation,” Qeios 2 (2020): WW04E6.2, 10.32388/WW04E6.2.

[36] M. S. R. Shawon , F. B. Hossain , G. Adhikary , et al., “Attitude Towards Diabetes and Social and Family Support Among Type 2 Diabetes Patients Attending a Tertiary‐Care Hospital in Bangladesh: A Cross‐Sectional Study,” BMC Research Notes 9 (2016): 286, 10.1186/s13104-016-2081-8. 27230084 PMC4881197

[37] A. A. Lee , J. D. Piette , M. Heisler , and A. M. Rosland , “Diabetes Distress and Glycemic Control: The Buffering Effect of Autonomy Support From Important Family Members and Friends,” Diabetes Care 41, no. 6 (2018): 1157–1163, 10.2337/dc17-2396. 29599295 PMC5961390

[38] E. Carduff , A. Finucane , M. Kendall , et al., “Understanding the Barriers to Identifying Carers of People With Advanced Illness in Primary Care: Triangulating Three Data Sources,” BMC Family Practice 15, no. 1 (2014): 48, 10.1186/1471-2296-15-48. 24690099 PMC3992158

[39] I. Røen , H. Stifoss‐Hanssen , G. Grande , S. Kaasa , K. Sand , and A. K. Knudsen , “Supporting Carers: Health Care Professionals in Need of System Improvements and Education—A Qualitative Study,” BMC Palliative care 18, no. 1 (2019): 58, 10.1186/s12904-019-0444-3. 31311536 PMC6636145

[40] M. Farquhar , “Assessing Carer Needs in Chronic Obstructive Pulmonary Disease,” Chronic respiratory disease 15, no. 1 (2018): 26–35, 10.1177/1479972317719086. 28685601 PMC5802659

